# 40. Impact of an Antimicrobial Intake Process within a Post-acute Medical System

**DOI:** 10.1093/ofid/ofab466.242

**Published:** 2021-12-04

**Authors:** Katherine M Shea, Segars Wayne, Jamie Stocker, Meredith Velez, Elizabeth Davis, Darrell Snider

**Affiliations:** Cardinal Health, Austin, TX

## Abstract

**Background:**

Implementation of antimicrobial stewardship programs (ASPs) within long-term acute care facilities (LTACs) is challenging due to limited resources and missing patient data from transferring facilities. In October 2018, an ASP was established within a 43-hopital system consisting of LTACs and rehabilitation hospitals. Despite the presence of a restricted antimicrobial policy, increased utilization was observed for five restricted antimicrobials. The system ASP committee implemented a multipronged approach to optimize utilization of these five agents. Investigators sought to assess the impact of an antimicrobial intake process on antimicrobial consumption.

**Methods:**

This was a retrospective analysis within a 43-hospital system of LTACs and rehabilitation hospitals, comparing use of five restricted antibiotics before (Jul19-Jun20) and after (Jul20-Apr21) implementation of a data-collection and system review process. An antibiotic intake form and process for review for five restricted antibiotics (ceftaroline, ceftazidime/avibactam, ceftolozane/tazobactam, fidaxomicin, meropenem/vaborbactam) was approved at the system ASP committee. The intake form consisted of a restricted antibiotic form, cultures and susceptibilities, physician notes, and other pertinent data. Any orders for the five antibiotics required completion of an intake form and submission to system ASP members for review and recommendations. Antibiotic consumption was measured in cost per acute patient day (cost/pd) using a 2-sided t-test.

**Results:**

Post-implementation, the five restricted antibiotics comprised 29.1% of the total antibiotic expenditure for the healthcare system compared to 35.6% pre-implementation. Ten months after program implementation, the total antibiotic cost/PD decreased 29.45% [(&12.02 ± 2.29) vs. (&8.48 ± 1.45); *p*=0.0003]. The cost/PD of the five restricted antibiotics decreased 42.52% [(&4.28 ± 1.09) vs. (&2.46 ± 0.99) ; *p*=0.0005].

**Conclusion:**

Implementation of an antimicrobial intake process within a post-acute medical system resulted in a significant reduction in antibiotic consumption for five targeted antibiotics as well as overall antibiotic expenditure.

**Disclosures:**

**All Authors**: No reported disclosures

